# The Rotation of Microrobot Simplifies 3D Control Inside Microchannels

**DOI:** 10.1038/s41598-017-18891-w

**Published:** 2018-01-11

**Authors:** Antoine Barbot, Dominique Decanini, Gilgueng Hwang

**Affiliations:** Laboratoire de Photonique et de Nanostructure, Centre National de la Recherche Scientifique, Marcoussis, 91460 France

## Abstract

This paper focuses on the control of rotating helical microrobots inside microchannels. We first use a 50 *μm* long and 5 *μm* in diameter helical robot to prove that the proximity of the channel walls create a perpendicular force on the robot. This force makes the robot orbit around the channel center line. We also demonstrate experimentally that this phenomenon simplifies the robot control by guiding it on a channel even if the robot propulsion is not perfectly aligned with the channel direction. We then use numerical simulations, validated by real experimental cases, to show different implications on the microrobot control of this orbiting phenomenon. First, the robot can be centered in 3D inside an in-plane microchannel only by controlling its horizontal direction (yaw angle). This means that a rotating microrobot can be precisely controlled along the center of a microfluidic channel only by using a standard 2D microscopy technology. Second, the robot horizontal (yaw) and vertical (pitch) directions can be controlled to follow a 3D evolving channel only with a 2D feedback. We believe this could lead to simplify imaging systems for the potential *in vivo* integration of such microrobots.

## Introduction

**Figure 1:**
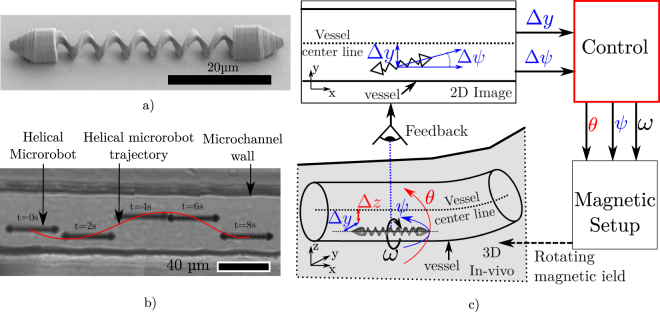
(**a**) SEM view of the helical microrobot used and modeled in this paper. (**b**) Photomontage experimental views showing the helicoidal trajectory of a helical microrobot in a microchannel. The medium is isopropyl alcohol. A video displaying this phenomenon is available as supplementary video 1. (**c**) Illustration of the loss of information due to 2D feedback. Δ*z* and Δ*y* are the distances between the robot and the channel center line, Δ*ψ* the misalignment between the robot and the channel orientation in the imaging plane. For the feedback, the information on *θ* and Δ*z* are not available. So the final objective of this paper is to propose a control of *θ* and *ψ* based on this partial feedback. All the angles are in the camera frame.

In the last decade research on mobile micrometric robots has grown interest, particularly in the field of biological and medical sciences. Existing proofs of concept have shown that such microrobots could perform various tasks such as: cell manipulation and enucleation^1^, selective gene transmission^2^, *in vivo* biopsy^3^ and force stimulation on a cell^4^. As these robots are too small to be embedded with motors and control mechanisms, one common solution is to propel them with a magnetic gradient that directly pulls them^5–8^ or with a homogenous rotating field that makes them rotate^9–13^. Compared to standard tethered tools these mobile robots have the advantage to access confined and closed environment. Microfluidic chips are a good example of such environment and several research articles including our previous work^14^ reported the integration and operation of microrobots inside them^4,15^. But as the surrounding dimensions reduce, controlling the robot without touching any surface becomes more difficult. For example, it would be relatively challenging to guide a 5 *μm* robot in a 20 *μm* diameter microchannel by following the center line of the channel to avoid any wall effect. To succeed this, the trivial solution is to increase the feedback resolution. However this leads to technical issues especially for estimating the robot altitude (i.e. the direction perpendicular to the imaging plane) as a complex equipment such as stereoscopy^8,16^, digital holographic^17^ or off-focus image analysis^18^ must be used. Therefore in this paper we propose to investigate how a natural phenomenon can be used to simplify the microrobot control in confined conditions such as a microchannel. We especially want to demonstrate that 2D feedback is sufficient to guide the microrobot on the channel center line. For this, we propose to use the phenomenon illustrated by the Fig. 1b with the helical microrobot pictured in Fig. 1a. Indeed we have experimentally observed that when a rotating robot is placed in a confined microchannel, its rotation provides a resulting force on the robot perpendicular to the channel direction. This force is due to the asymmetry of the boundary conditions in the flow produced by the robot rotation. It results in the orbiting of the robot around the channel center line while it propels itself through the channel. We will refer to this phenomenon in the following as the orbiting phenomenon. A similar focusing phenomenon in the channel cross-section has been described^19^ and theorized^20^ for passive objects at low Reynolds number. Inertia focusing is used in microfluidics to center^21^ and sort^22^ particles. The particular dynamic of a microrobot in channel has recently being investigate at the millimetric scale and a similar orbiting phenomenon was reported^23^. Different microswimmer trajectories were also theoretically investigate in this case^24^. Therefore our work focus on how the microrobot control can benefit from this phenomenon.

To prove that the control of rotating microrobots inside microchannel is possible, this paper proposes a physical model that takes account of this orbiting phenomenon. The main challenge of such control is to follow the channel center both in the horizontal plane corresponding to the optical plane as well as in altitude. The Fig. 1c illustrates this challenge and shows that the information on Δ*Z* (The direction perpendicular to the optical plane) and the orientation of the channel on *θ* are not available. The question to answer is then: “How can we control the angle *θ* of the robot with only Δ*ψ* and Δ*y* (both available by a 2D imaging system) while guaranteeing that the robot follows the centerline of the channel”?

In the case of a microfluidic chip, channels evolve in a single plane, therefore the orientation of the channel on *θ* is null which slightly simplifies the problem. However another goal of this paper is to propose potential control toward *in-vivo* integration in blood and lymphatic vessels which evolve in 3D and where 2D feedback does not allow to know *θ*. Classical *in-vivo* imaging methods based on MRI, X-ray and ultrasound, allow 3D imaging modes, but it naturally comes with a decrease in resolution or an increase in processing time compared to 2D modes. So a control strategy working with only 2D feedback information could simplify the imaging requirement to achieve real time control of microrobot. Therefore we believe that such control could contribute in lowering the technical gap toward *in-vivo* microrobot medical mission. The results of this paper are organized as follows: First we present our numerical model and validate it by comparing some of its results with real experimental cases; Second, we use this model to prove that the orbiting phenomenon enables the microrobot to follow the channel by avoiding contact with the surface even if the robot is misaligned with the channel direction; Third, we demonstrate that only the control of the *ψ* angle is enough to center the helical robot on the microchannel center if this channel evolves in a plane; Finally, we demonstrate that thanks to the orbiting phenomenon, the two directions *ψ* and *θ* of the robot can both be controlled in a 3D evolving microchannel by using only Δ*y* and Δ*ψ* measurements available by 2D feedback. For details on the control and integration of the microrobot inside a microfluidic chip we refer to our previous publication^14^.

## Results

### Numerical model and experimental validation

Before making a model of the helical microrobot movement, we need to make several assumptions. To justify these hypotheses, we consider a model case with the maximum encountered speed in our experiments. Therefore, we consider a helical robot with a diameter of 6 *μm* and a length of 50 *μm*, rotating at 200 *Hz* and having a maximum speed of 200 *μm*·*s* ^−1^ in isopropyl alcohol. In experimental conditions we are always below all these values. By considering this scenario, we can guarantee that the following hypotheses are always justified:

- The fluid is incompressible. This assumption is usually made in fluid simulation when the Mach number is below 0.2^25^. This number is equal to the maximum speed of the fluid divided by the sound speed in this medium. By considering the maximum fluid speed at the boundary of the helical microrobot, the Mach number is less than 10^−6^.
- The fluid inertia is negligible. In general, a fluid is governed by its inertia and viscosity. The Reynolds number is the ratio between the inertia and the viscosity terms: ${\mathcal R} =\frac{LV\rho }{\nu }$, where *L* is the typical length of the object, *V* the typical speed, *ρ* the fluid density and *ν* the fluid dynamic viscosity. In our theoretical case presented above, ${\mathcal R} =2\cdot {10}^{-2}$ which is largely below 1 and therefore justifies neglecting the inertia of the fluid in our simulation.
- The helical microrobot inertia is negligible. By using a rough estimation of the robot mass and drag coefficient in the speed configuration presented above, it will take approximately 20 *μs* and travels 1 *nm* to stop if the propulsive force is suddenly stopped. We consider both these values small and we neglect the robot inertia. Thus it means that the speed of the robot in liquid is proportional to the sum of external forces at any times.

**Figure 2:**
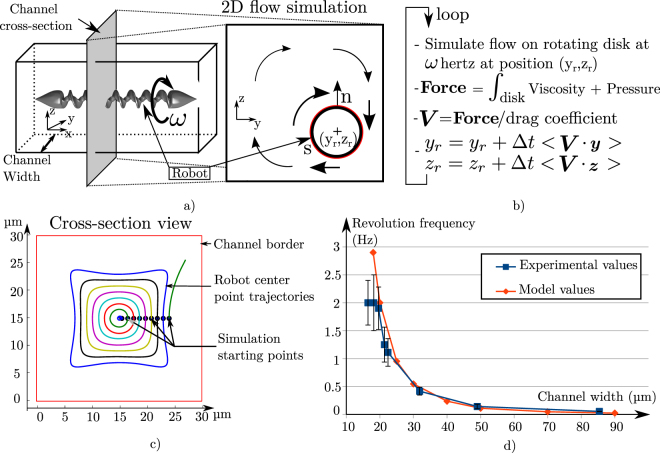
Cross-section channel trajectory simulations. (**a**) Shows how only the cross-section of the channel at the helical microrobot position is considered for the simulation. (**b**) Is the pseudo-code of the simulation process of the robot trajectory on this moving cross-section. (**c**) Shows results of trajectory simulations for different starting points on a 30 *μm* width square channel. (time step = 0.05 s). (**d**) Shows comparison of the revolution frequency (corresponding to the time for the robot to come back to its starting point on the cross-section) with the width of the channel. Results are shown both for simulation and model.

In this model we propose to simulate the helical microrobot position in the cross-section of the channel with numerical simulations. To simplify the problem, we consider the robot as an infinite cylinder perfectly aligned with an infinite microchannel. A cylinder does not take into account the corkscrew shape and therefore does not simulate the flow parallel to the helical microrobot producing propulsion. However we make this strong assumption as we are not interested in this propulsive flow but only by the flow in the cross-sectional plane. Indeed, this flow is the one that can produce a fluid drag perpendicular to the RTS and the channel direction. The decoupling of these two problems is justified by the low Reynolds conditions that guarantees the linear addition of the flow solutions. Thanks to this assumption, we consider only the 2D problem in the cut plane defined by the microchannel cross-section as shown on Fig. 2a. In the numerical simulations, we solve the Stokes equation given by:

1 $$\nu {\nabla }^{2}u-\nabla P=\mathrm{0,}$$

with *u* the speed of the flow, *P* the pressure and *ν* the dynamic viscosity.

The boundary conditions are such that the flow speed is null on the microchannel wall and is equal to the RTS rotation speed on its surface. We used a finite element method simulation software (Freefem++^26^) to perform the flow simulations and a Python script to integrate the trajectory on the channel cross-section. As the Reynolds number of the flow is small, the flow solution and associated pressure field evolve linearly with the boundary conditions i.e. the rotating frequency of the robot. We therefore fix the rotating frequency to 100 Hz as we don’t need to investigate the influence of this parameter.

The Fig. 2b shows the pseudo-code of this simulation. At each step the flow is solved to integrate the value of the viscous and pressure drag on the robot. The lateral speed is obtained by dividing this force by the lateral drag coefficient of the robot which is determined experimentally. Finally, we extrapolate the new position of the robot from this speed after a small time step. The Fig. 2c shows the trajectories resulting from this simulation for different starting point. Here the corresponding microchannel is a straight microchannel with a perfectly aligned microrobot. These trajectories show that the robot orbits around the channel center and returns after one turn to its starting position in the channel cross-section. Moreover supplementary material Section Supplementary Information Section A shows that the different frequencies of these orbits are almost equal for any starting points regardless of the distance to the channel center.

Note that the force from the pressure is approximately two times more important than the viscous one. supplementary material Section Supplementary Information Section C presents two force profiles on a line of the channel cross section. Moreover as the flow distribution is entirely fixed by the boundaries condition, it not depends on the viscosity. Therefore thanks to the equation 1, the pressure field values evolve linearly with the viscosity. The viscous force due to the microrobot rotation also evolve linearly with the viscosity, we support this demonstration by some simulation run at different viscosity on supplementary material Section Supplementary Information Section C. Therefore as the fluid drag coefficient also linearly dependent on the viscosity, the integration of the microrobot position will be the same for medium with different viscosity. We can see that the orbiting frequencies highly depend on the channel width. Therefore we use this evolution to validate our model. The Fig. 2c displays the evolution of this orbiting frequency with the channel width obtained both by real experiments and by our model. As the model corresponds to the reality and predicts well the increase of the frequency with the channel width reduction, we conclude that it is valid and can predict the position of a rotating helical microrobot in the channel cross-section.

To simplify the analysis of the rotating phenomena and its control, we limit in this paper to channels with a depth-to-width ratio of 1 (i.e. with a square section). For different ratios, the rotating phenomena still exist but two different orbiting center in the channel cross section exist. They are located along the longest center line. In this case the control demonstrated in the following part is still possible around one of the two center points. More details of this case are presented in section Supplementary Information Section F.

### Misalignment between the Microrobot and the Microchannel

**Figure 3:**
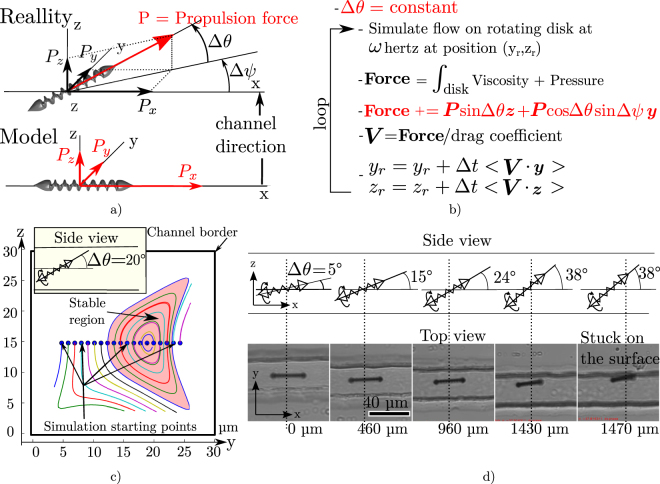
Simulations and experiments of *θ* misalignment. (**a**) Shows that we approximate a misaligned robot with the channel by an aligned robot with some perpendicular thrust force component. (**b**) Is the pseudo-code of the simulation process, the updated part is highlighted in red. (**c**) Show cross-section trajectories for a misalignment of 20° between the robot and the channel. (**d**) Shows an experimental demonstration where the pitch angle is increased up to 38° before touching the channel wall in a 40 *μm* square channel. Supplementary video 2 displays this phenomenon.

As we demonstrated the global validity of our model, we will now use it to prove that the microrobot rotation simplifies its control along a microchannel. Indeed, we saw experimentally that helical microrobots could be guided through a microchannel even if a misalignment between the robot and channel direction is present. This is shown by Fig. 3d where a misalignment of 38° on Δ*θ* was needed to make the robot touch the channel surface.

Figure 3a shows how we propose to model the misalignment of the microrobot with the channel. For this, we consider that a misaligned robot has the same geometry as an aligned one, however, the lateral forces corresponding to the misaligned components of the robot thrust, are added to the simulation results. This assumption allows us to keep the same model in 2D for the flow simulations. It is only valid for small misalignment and we believe that for large one the results can be considered only qualitatively. Figure 3b shows how these changes are applied in the simulation code by highlighting the new part of the pseudo-code in red. This new model allows us to simulate the misalignment of the robot with the channel. We therefore chose to simulate the trajectories on the cross-section of the channel with different Δ*θ* values. Figure 3c displays this result for Δ*θ* = 20 in a 30 *μm* width channel. On this result we can see that this misalignment deforms the trajectories but that part of them are still stable and avoid channel walls. This means that for any starting point inside this region, the robot will be guided along the channel despite its misalignment. This region get smaller as the misalignment angle is increased. It also reduces compare to the channel size, for bigger channels as the orbiting phenomenon and its corresponding viscous and pressure force became less important. Indeed, the maximum misalignment angle to sustain a stable region for 90 *μm* channel is 4°. We refer to supplementary material Section Supplementary Information Section D for simulation results showing four different Δ*θ* for four different channel width values (18, 25, 30 and 50 *μm*). Therefore we can conclude that the rotation of the robot induces a force that guides it along the channel even if the thrusting force is not exactly aligned with this channel. As it is due to the orbiting of the robot in the cross-channel, it can only happen for channel width dimension larger than 1.5 times the robot diameter. This phenomenon starts to be negligible for dimension larger than 20 times the robot diameters for a 100* Hz* rotation frequency.

### *ψ* controlled, *θ* = constant, in straight channel

**Figure 4:**
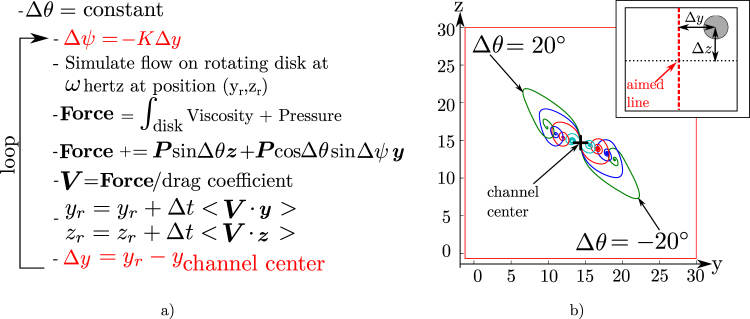
*ψ* Controlled at different *θ*. (**a**) Shows the pseudo-code. A proportional controller controls the value of *ψ* by using Δ*y*, the distance between the robot and the channel center on the y-axis. (**b**) Shows the result of trajectory simulations for different value of *θ* with *K* = 0.25*Rad*·*μm* ^−1^. This result shows that a misalignment on *θ* has an impact on Δ*y* and therefore that Δ*y* could be used to control *θ*.

The orbiting phenomenon guarantees that a misaligned propulsion can guide a robot on a channel. However we showed that if this misalignment becomes too important the robot can touch the wall. Moreover it doesn’t guarantee that the robot is maintained close to channel center. However this center is the best place to be robust to brutal change in channels geometry or orientation as not every starting point leads to a stable path. Therefore we propose to use our model to develop a control strategy for the microrobot to follow the channel center line. As we explained in the introduction, developing a 3D feedback system has disadvantages: it can be an important constraint in a design, it can increase the feedback time delay and it can reduce the feedback resolution. This is why we want to propose a solution only based on a 2D feedback. This means that we can only measure Δ*y* which is the distance of the robot to the channel center projected on the y-axis. Δ*z* is not measurable. Therefore only the *ψ* angle of the robot can be controlled resulting in a control aiming a plane and not a line. Figure 4a shows what this control changes in our pseudo-code as we choose to control the robot *ψ*_*r*_ angle by a simple proportional law:

2 $${\psi }_{r}={\psi }_{c}-K{\rm{\Delta }}y,$$

with *ψ*_*c*_ the yaw angle of the channel and *K* a constant. Figure 4b shows the result of this simulation for different misalignments on *θ*. First we can see that only the control of the *ψ* angle is enough to focus the robot on a point relatively close to the center depending on Δ*θ*. This first result is especially interesting for the control of the robot in 2D evolving microchannel such as for example the channel on a microfluidic chip. Indeed in this case Δ*θ* is null and it means that the simple control of *ψ* is sufficient to center in 3D the rotating robot in the middle of the channel. Therefore a simple microscope without any altitude tracking provides enough feedback to perform this 3D centering as the control of *ψ* makes Δ*y* converge to zero as well as Δ*z* thanks to the orbiting phenomenon. The second interesting result is that the focusing point position changes as Δ*θ* changes. Moreover this change is measurable on the y-axis. Therefore it means that Δ*y* could be used as feedback for a potential control of Δ*θ*. This is what we test with our simulation in the following subsection.

### *ψ* controlled, *θ* controlled, in curved channel

As we just demonstrate, the orbiting phenomenon allows to control a microrobot on a channel even with small misalignment exists. Therefore controlling *θ* is only useful in case of a channel with direction evolving in 3D such as *in vivo* vessels. For this, the main problematic here is that a 2D feedback can only give the distance from the robot to the channel center on one axis, the other one cannot be measured. Therefore there is no parameter to control the *ψ* angle in a straightforward way. However, thanks the result of the Fig. 4, we see that a misalignment on *θ* as an impact on Δ*y*. Therefore thanks to the orbiting phenomena, Δ*y* is a good candidate to control as well *θ* in addition of *ψ*. This could allow a 3D control on the channel center only thanks to 2D feedback.

**Figure 5:**
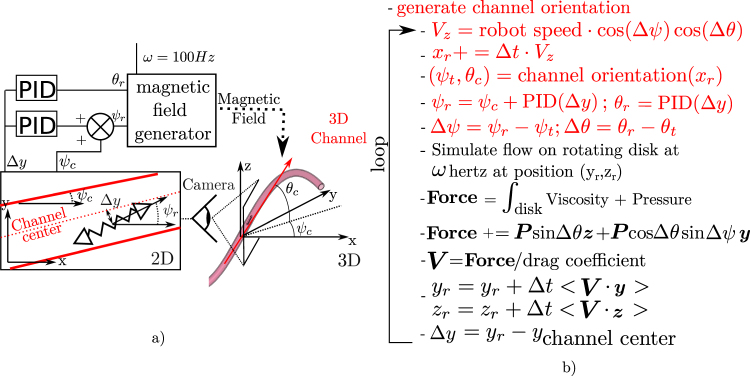
Principle of the Helical microrobot simulation on a channel evolving in 3D. (**a**) Is a schematic of the control loop (**b**) Present the simulation pseudo-code, the modifications are highlighted in red.

**Figure 6:**
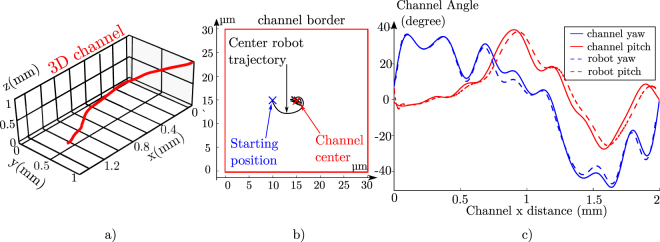
Result of the helical microrobot simulation on a channel evolving in 3D. (**a**) Displays a 3D view of a randomly generated channel. (**b**) Shows the helical microrobot position in the channel cross-section during its control (*ψ* PID: *P* = 0.04*Rad*·*μm* ^−1^, *I* = 0.01*Rad*·*μm* ^−1^ ⋅ *s* ^1^; *θ* PID: *P* = 0.02*Rad*·*μm* ^−1^, *I* = 0.1*Rad*·*μm* ^−1^ *s* ^1^, *D* = 0.02*Rad*·*μm* ^−1^ *s* ^−1^ (**c**) Shows the evolution of the yaw and pitch of the channel and robot in the camera frame.

In order to see if Δ*y* information is sufficient to control both *ψ* and *θ*, we decide to simulate a 3D evolving channel and to make our robot evolve in it. Figure 5a illustrates the control feedback that we are modeling and 5 b) shows how the simulation code is adapted. The goal of this control is that *ψ*_*r*_ and *θ*_*r*_, the robot angle values, follow the channel ones: *ψ*_*c*_ and *θ*_*c*_. A complete work flow of the simulation process is presented in supplementary material Section Supplementary Information Section D. To control *θ*_*r*_ (the robot pitch) we use a Proportional Integrative Derivative (PID) corrector feed with Δ*y*. Indeed as no direct measurement of *θ*_*c*_ (the channel pitch) is possible the integrative gain is essential to converge on a solution with a pitch angle different from 0. A PID controller is also used for *ψ*_*r*_ as it proves to be more efficient than a simple proportional corrector in our different simulations with certain configurations. Figure 6a displays a 3D representation of the simulated channels. Figure 6b shows the evolution of the helical robot position on the moving cross-section of the channel during the simulation. We see that the robot quickly converges and stays around the center of the channel during all the simulation. Finally, Fig. 6c displays the evolution of the channel and robot angles. Here the robot angles follow the channel angles well during all the trajectory. A small delay is present on *θ*, this delay is due to the low proportional corrector value. This one is needed because the Δ*θ* value is only measurable as the position start to converge due to the control on *ψ*. However the two microrobot angles successfully follow the channel angle. This demonstrates that the orbiting phenomena could allow microrobot control in a 3D microchannel by only using a 2D feedback system. supplementary material Section Supplementary Information Section E shows results for four other channel widths (18, 25, 40 and 50 *μm*) and illustrates the difficulties of this control as the channel dimensions increases. As for the robustness to misalignment, the control in 3D is simpler on smaller channel as the orbiting force is larger and frequency higher. Indeed we could not find proper PID corrector values in channel cases with dimensions above 50 *μm* which is 10 time the robot diameter. In real case smaller channel dimension could also reveal some difficulty depending on the quality of the feedback. Indeed a noisy feedback could prevent highly sensitive gain for PID tuning and result in triggering instability. The value of the gain corrector also needs to be adapted depending on the channel width. In a real case with an evolving size of channel, this adaptation could be achieved by measuring the orbiting frequency as is correlated to the channel dimension (see Fig. 2d).

We can note that in our model we consider a robot with a neutral buoyancy and therefore we neglect the impact of the gravity force. In real experiments, this force which depends on the liquid density, acts as a perpendicular parasite force in channels with a horizontal component. So another advantage of controlling *θ* angle with a PID feed with Δ*y*, is to solve the gravity compensation at the same time as the misalignment problem. Indeed to maintain the microrobot center in the channel, *θ* will converge to a solution presenting a small misalignment with the channel direction that compensates the gravity effects.

### Trajectory control in Y-junction microchannel

**Figure 7:**
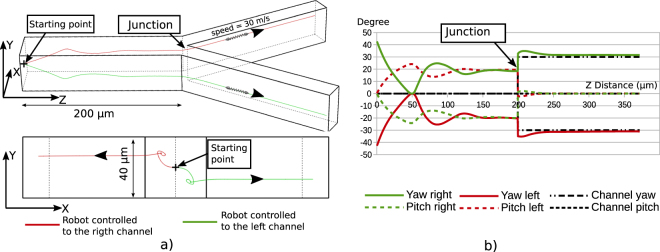
Simulations of the helical microrobot trajectories through a Y type channel junction. The main channel has a square section of 50 *μ* m side. This channel splits in two similar rectangular section with 50 *μ* m over 25 *μ* m. (**a**) Show trajectories base on a 2D image feedback control using the position of the robot on the x coordinate to control the yaw and pitch angle. Similar simulations are made to simulate the control of the robot in the left channel. (**b**) Shows the evolution of the yaw and pitch angle on these two trajectories. Note that after the junction the yaw angle is different as the channel has a different orientations.

The rotation phenomenon also simplifies the trajectory control in a Y-type junction channel. Particularly it can be used to select in which channel the microrobot will go after the junction. As for the other presented control, only a 2D based control can be used. However instead of aiming at the channel center, the yaw angle is controlled by the distance of the robot to a point at the left or the right of the channel center. The Fig. 7 shows the simulation of this control. In this case, if we want the robot to go in the left channel after the junction, the aimed x position will be 25 *μ* m to the left of the center. This new trajectory will place the robot in the correct position to continue in the left channel. After the junction the control can be reset to the center of the new channel to assure a safe distance from the channel wall in the smaller channel.

The Fig. 7a Show microrobots trajectories resulting from two different control. Similar simulations are made to simulate the control of the robot going either in the right or the left channel. For the y coordinate, the robot position is attracted to a natural stable value. This value is changing depending on the x controlled value. Therefore, the two different trajectories have different y coordinate. After the junction, the channels have not a square section so two stable positions exist as we explain in the Supplementary information section F. Therefore the robot continues with two different y coordinates on the left and on the right channel which correspond to the two different stable positions. The Fig. 7b show the evolution of the microrobot angle for the left and right trajectories. Even before the junction the pitch angle is controlled by the distance on x to the channel center. This allows the robot to compensate for the force on the y axis. This is necessary to get a stable control position different from the center.

The simulations are made in two steps. In the first step, the control of the robot before the junction is simulated. Then in the second step the position and orientation of the robot at the last simulation point are saved and pass to a new simulation using the geometry after the junction. The PID control parameters remain unchanged between the two simulations and the channel change of orientation is taken into account. The limit of this model is to pass brutally from one geometry to another, however, we can see that the positioning of the robot before the simulation lead it to a position close to the stable position after the junction. Therefore, at the junction, the influence of the post-junction geometry should have only a little impact.

Supplementary Information Section G, present another bifurcation example where the bifurcation is not in plane.

## Conclusion

In this article, we showed that an orbiting phenomenon around the channel center happens when a rotating microrobot evolves inside a microchannel. This phenomenon makes the robot rotate around the channel center line. We proved experimentally that this phenomenon was noticeable for channels dimension from 1.5 times to 20 times the microrobot diameter at a 100* Hz* rotation frequency in low Reynolds flow condition. We also experimentally demonstrated that this phenomenon simplifies microrobots control by guiding the through the channel even if it is misaligned with the channel direction. Therefore a first conclusion of this work is that the rotation of a microrobot simplifies its control in a microchannel by avoiding contact with the surface. We then proposed a 2D model of this phenomenon which was validated with experimental results. This simulation first demonstrated that the orbiting frequency was only depending on the channel dimension and not on the position of the robot in the channel. This opens perspectives to measure the channel dimension based on this orbiting frequency. Then, we used this simulation to prove that the orbiting phenomenon facilitates the microrobots control. We first proved that only the control of the yaw (*ψ*) angle is sufficient to focus a rotating microrobot on a channel center for a channel evolving in a horizontal plane. Indeed the orbiting phenomenon ensures that the control of the horizontal coordinates to the channel center implies the control of the altitude (i.e. the dimension perpendicular to the horizontal plane) to the channel center altitude. The conclusion is that a simple 2D image feedback by a microscope is sufficient to guide a rotating microrobot in a standard microfluidic channel. In this case the robot will be focused on the channel center line and contact with the channel wall will be prevented. Finally, we showed by simulation that the information of the robot distance to the channel center projected on one coordinate is sufficient to control both the robot yaw (*ψ*) and pitch (*θ*) to follow the channel direction. This is again due to the orbiting phenomenon that leads to different focusing positions on the horizontal plane for different misalignment angles on the pitch (*θ*). This implies that only a 2D feedback could be used to successfully guide a microrobot in a 3D evolving channel having the same geometry as blood or lymphatic vessels. We also demonstrated how it could help to guide microrobot through channel junction But if we consider *in-vivo* condition, the non-constant flow of the bloodstream and the feedback limitation of medical imaging systems are serious challenges to the microrobot control. However we believe that demonstrating and modeling control strategy in a simple environment, easier to model and understand, is the first step in order to develop the control in more complex environments such as *in-vivo* ones. Therefore further research should first focus on proving the proposed 3D control strategy experimentally in well controlled and understood microfluidic environment. Then more real conditions could be considered by testing non-homogenous flow profile in the channel as well as non-Newtonian fluid closer to a biological one. However despite its simplicity we think that the proposed 2D cross-section base model in this article is enough to demonstrate the potential of the orbiting phenomena to help control of microrobots. Indeed thanks to the linearity of the flow solutions at low Reynolds number, we can at least guarantee that this phenomenon will still exist and in the same intensity for any flow profile.

## Materials and Methods

### Helical Microrobot Fabrication

The helical microrobots are made in resist (IPG), by using two-photon lithography with the commercialized “nanoscribe” machine. To actuate them with a magnetic field, a Physical Vapor Deposition (PVD) process (Plassys MEB550SL) is used. It first deposes a 20 nm chrome layer for adhesion at both 0° and 75° inclination. Then it deposes a 100 nm nickel layer both at 0° and 75° inclinations. The microrobots are detached from the substrate selectively by a tungsten tip and then integrated inside the microfluidic chip. The intensity of the homogenous rotating field that makes them rotate is around 10 mT.

### Microfluific Fabrication

The microfluidic chip is made in Polydimethylsiloxane (PDMS). For this, we first made a mold in Poly-methyl methacrylate (PMMA) with a micromilling machine. The maximum resolution of the milling process is 1 *μm*. The unreticulated PDMS is mixed with the reticulating agent and pour on the mold. It is left 50 minutes to reticulate at 70 °C. Then we remove the solidified PDMS from the mold, make holes for fluidic injections and bound it to a glass substrate by using a 0_2_ plasma. The final chip is then stored at least 12 hours in 70 °C for PDMS to completely finish its reticulation.

### Computational Fluid dynamics (CFD) details

The fluid simulations are made using FreeFem++ software^26^. we solve the Stokes equation in the 2D channel cross-section which is given by:

3 $$\nu {\nabla }^{2}u-\nabla P=\mathrm{0,}$$

with *u* the speed of the flow, *P* the pressure and *ν* the dynamic viscosity.

The boundary conditions are such that the flow speed is null on the microchannel wall and is equal to the RTS rotation speed on its surface. The RTS section is modeled by a circle with a 2.5 *μm* radius. In order to avoid the flow simulation at each timestep of our simulation, these simulations are made in a pre-process step. For this, we create a 50 by 50 grid that equally divides the channel cross-section and the simulation is run with the RTS positioned at each node. For each of these simulations we integrate the fluid drag on the RTS surface as well as the pressure force. This integral is computed inside FreeFem++. Therefore during the trajectory simulation, the fluid force is linearly interpolated on the grid for each robot. This method is justified by the low Reynolds conditions that guaranty the non-dependency of a solution with time. Supplementary Information Section H presents a manual to use the source code to reproduce the results present in this paper. The Flow simulation source code is available on Supplementary Information Section H.1 and the trajectory integration and control on Supplementary Information Section H.2.

## Electronic supplementary material

Video 1

Video 2

Supplementary document
