# Supplementary material for: The Rotation of Microrobot Simplifies 3D Control Inside Microchannels
Source: Sci Rep. 2018 Jan 11;8:438. doi: 10.1038/s41598-017-18891-w (PMC5765130; doi:10.1038/s41598-017-18891-w)
Supplement: Supplementary file 3 — Supplementary document [file 41598_2017_18891_MOESM3_ESM.pdf]

Supplementary Information

The Rotation of Microrobot Simplifies 3D Control Inside Microchannels

Antoine Barbot[1], **Dominique Decanini**[1], **and Gilgueng Hwang**[1,*]

[*]gilgueng.hwang@lpn.cnrs.fr
[1]Laboratoire de Photonique et de Nanostructure, Centre National de la Recherche Scientifique, Marcoussis, 91460, France

ABSTRACT

This document presents supplementary information for the paper : The Rotation of Microrobot Simplifies 3D Control Inside Microchannels. Extended result of the numerical simulations and explanation of the model are presented. The influence of the channel aspect ratio and of the fluid viscosity is investigating. Finally, thanks to a source code and a practical manual one can reproduce the simulation presented in the main article.

A Low position dependency of the orbiting rotating frequency

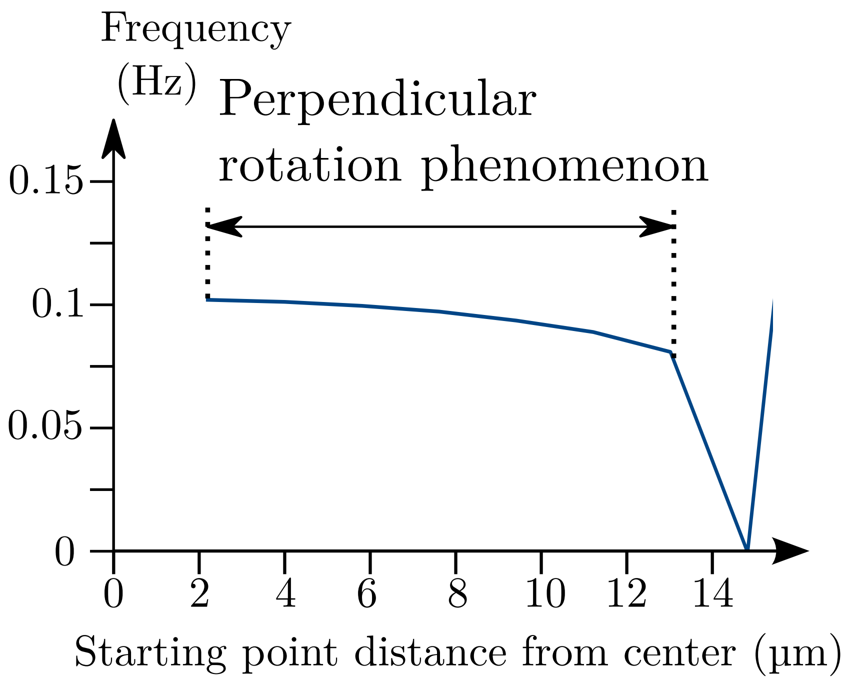

Figure A.1. Frequency of the orbiting phenomenon for different distance from the channel center. This result proves that the orbiting phenomenon has approximately a constant frequency for a given channel width.

B Complete 3D evolving channel work flow

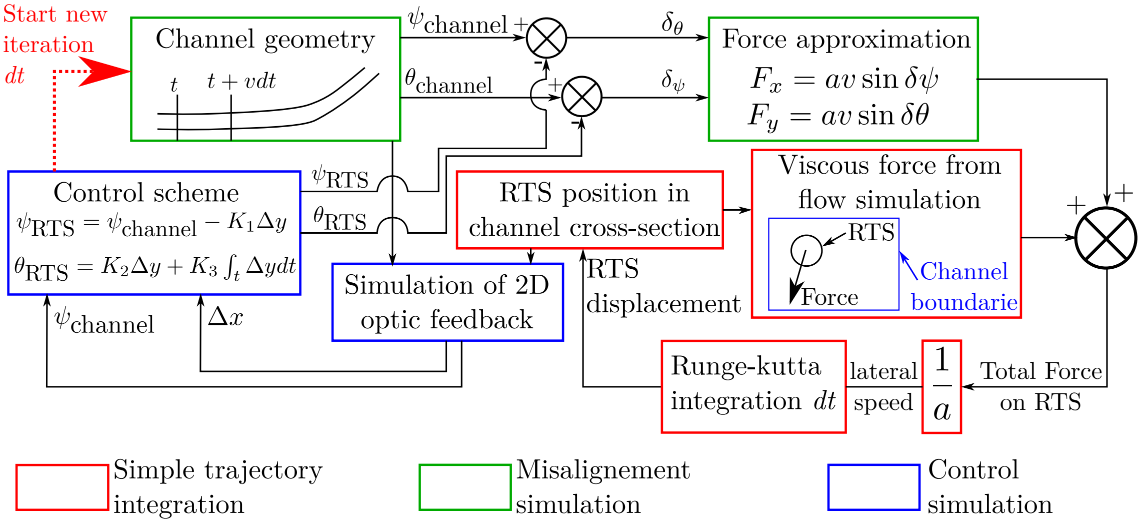

Figure B.1. Complete Work flow of the control simulation in 3D evolving micro channel

C Pressure and viscous force evolution

Four simulations with different fluid viscosity on a 30μm channel show that the total force applied on the robot evolves linearly with the viscosity. The graphs presented in Figures C.1 b) c) represent the evolution of the viscous and pressure force along the center line displayed by the Figure C.1 a). The simulations are made with different viscosity to illustrate the linear evolution of both forces with this parameter. The figure C.1 d) shows this linearity for the point on the center line with y = 8 μm.

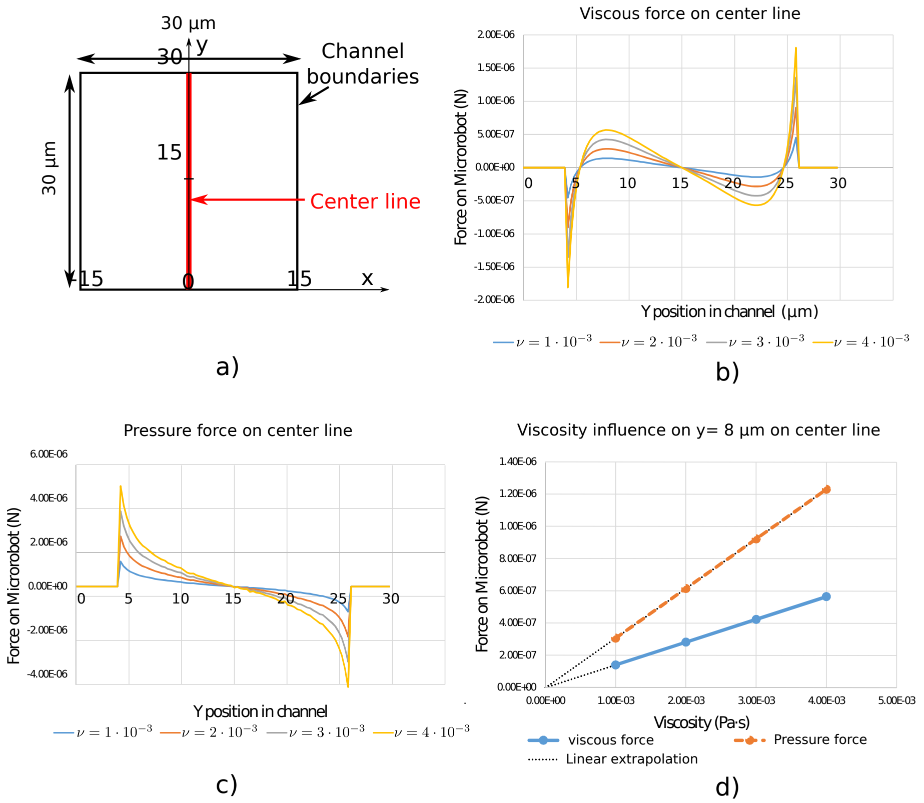

Figure C.1. Pressure and viscous force evolution on the center line of the cross section. a) shows the center line on the channel cross section. b) and c) respectively show the evolution of the viscous and pressure force on the rotating microrobot at different positions on this line. d) shows the linear evolution of both viscous and pressure line with the viscosity in a particular position of the channel cross-section.

D Simulation results: varying misalignement angle in different channel width

Figure D.1 illustrates that the orbiting phenomenon forces decrease with the channel dimension. Therefore larger channels are less sensible to misalignment of the robot.

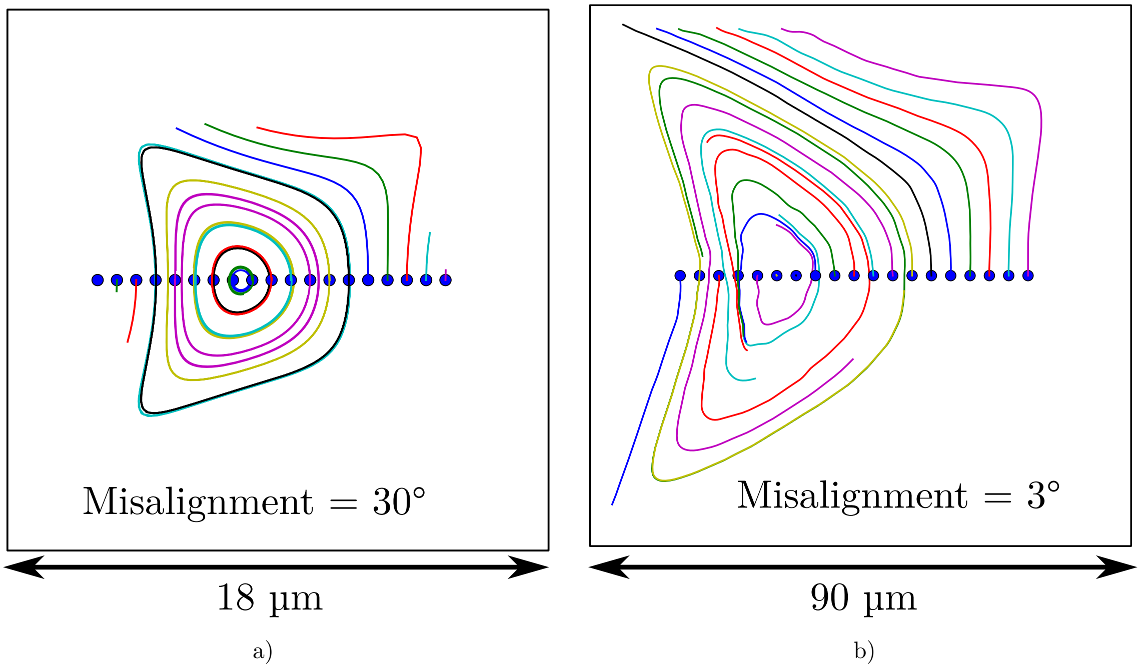

Figure D.1. Multiple trajectory with different starting points. a) for 30° misalignment in a 18 μm straight square channel b)for 3° misalignment in 90 μm straight square channel.

E Simulation results: 3D control in different channel width

On the following we present simulation of the 3D control algorithm in channel size varying from 18 μm to 50 μm. Above 50 μm the orbiting phenomenon is too weak and we could not find a proper tuning of the control that avoids touching the channel surface. For each case we find a working solution for the PIDs tuning. These solutions were found by manual trial and error because of the non-linearity and mutual dependency of yaw and pitch angle control. Therefore we do not pretend these coefficients to be the optimal ones but just working solutions. Nonetheless we can see that working values for the corrector decreases with the increase of the channel dimension.

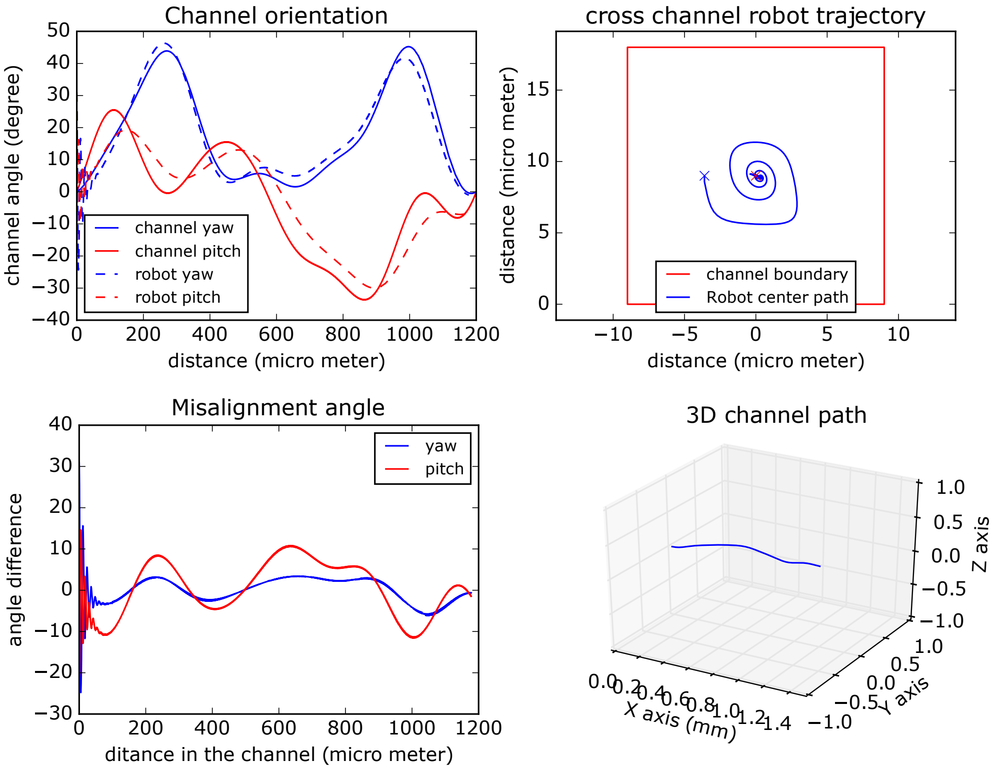

Figure E.1. Trajectory simulation in 18 μm square section channel. Control parameters: ψ PID: $P = 0.15 Rad \cdot \mu m^{-1}$, $I = 0 Rad \cdot \mu m^{-1} \cdot s^{1}$,$D = 0 Rad \cdot \mu m^{-1} \cdot s^{-1}$; θ PID: $P = 0.1 Rad \cdot \mu m^{-1}$,$I = 0.3 Rad \cdot \mu m^{-1} \cdot s^{1}$,$D = 0.02 Rad \cdot \mu m^{-1} \cdot s^{-1}$

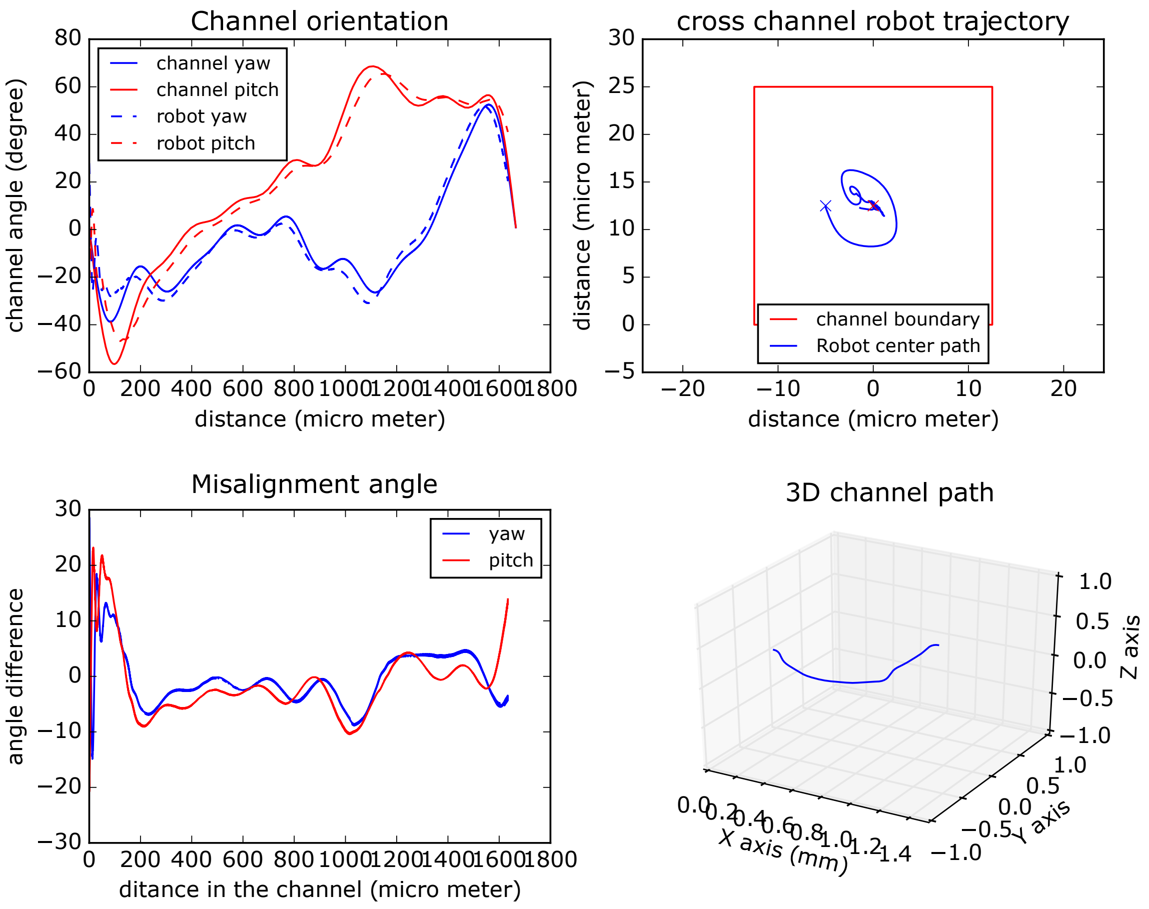

Figure E.2. Trajectory simulation in 25 μm square section channel.Control parameters: ψ PID: $P = 0.1 Rad \cdot \mu m^{-1}$, $I = 0 Rad \cdot \mu m^{-1} \cdot s^{1}$,$D = 0 Rad \cdot \mu m^{-1} \cdot s^{-1}$; θ PID: $P = 0.075 Rad \cdot \mu m^{-1}$,$I = 0.15 Rad \cdot \mu m^{-1} \cdot s^{1}$,$D = 0 Rad \cdot \mu m^{-1} \cdot s^{-1}$

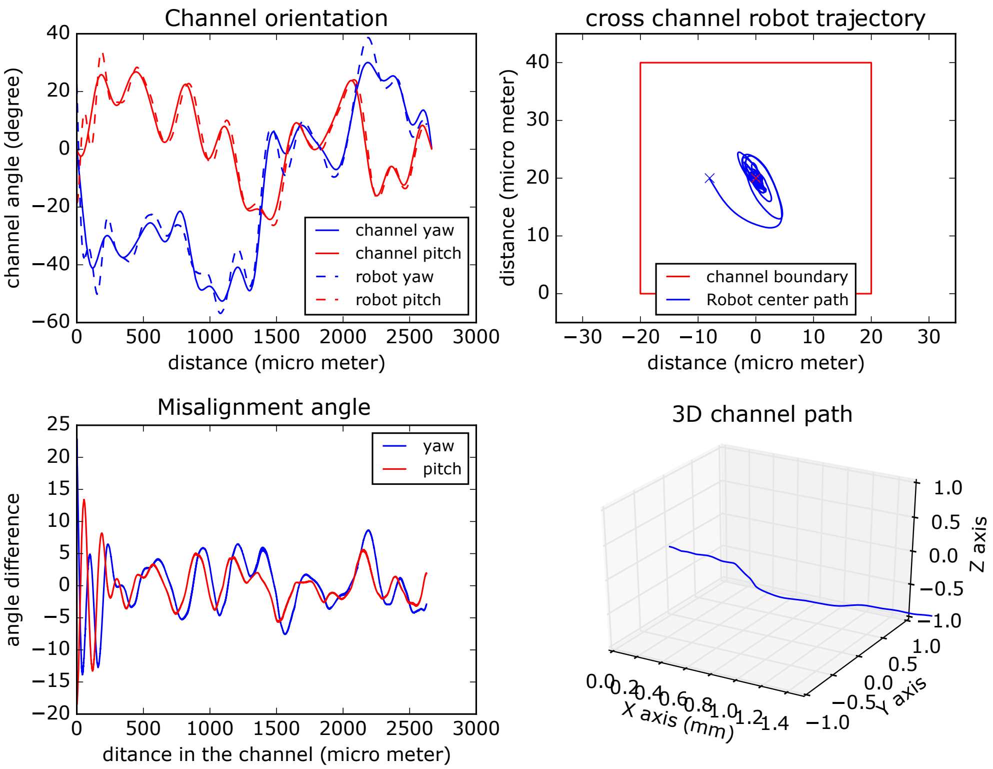

Figure E.3. Trajectory simulation in 40 μm square section channel.Control parameters: ψ PID: $P = 0.05 Rad \cdot \mu m^{-1}$, $I = 0 Rad \cdot \mu m^{-1} \cdot s^{1}$,$D = 0 Rad \cdot \mu m^{-1} \cdot s^{-1}$; θ PID: $P = 0.04 Rad \cdot \mu m^{-1}$,$I = 0.08 Rad \cdot \mu m^{-1} \cdot s^{1}$,$D = 0 Rad \cdot \mu m^{-1} \cdot s^{-1}$

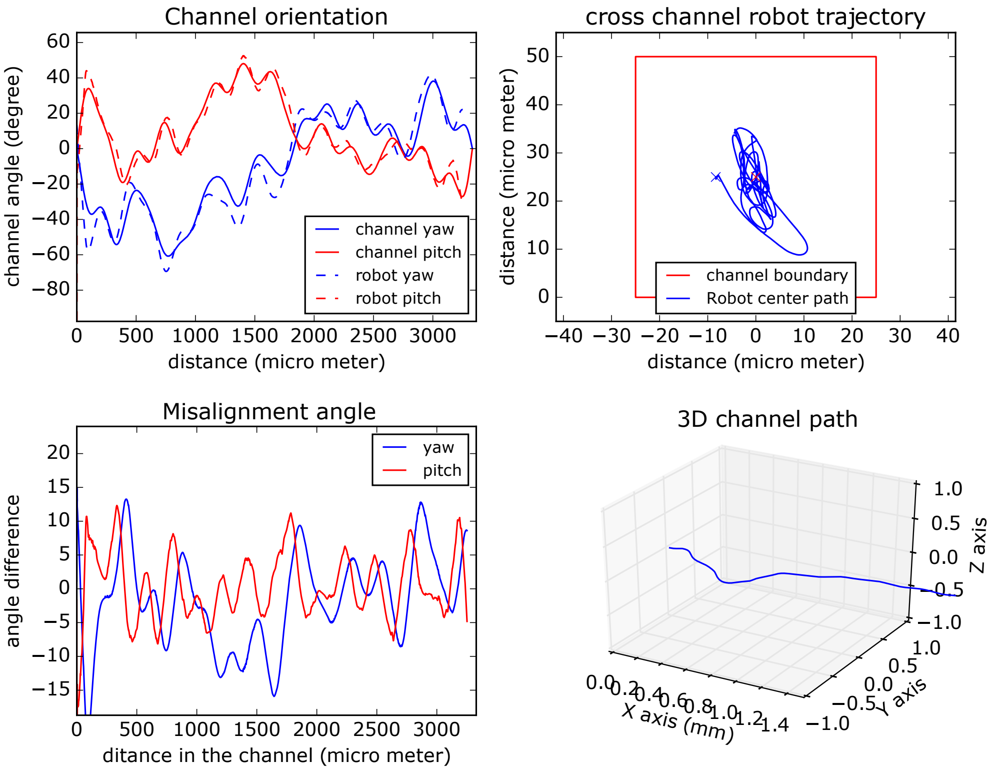

Figure E.4. Trajectory simulation in 50 μm square section channel. Control parameters: ψ PID: $P = 0.03 Rad.\mu m^{-1}$, $I = 0.01 Rad.\mu m^{-1}.s^{1}$,$D = 0 Rad.\mu m^{-1}.s^{-1}$; θ PID: $P = 0.05 Rad.\mu m^{-1}$,$I = 0.04 Rad.\mu m^{-1}.s^{1}$,$D = 0.02 Rad.\mu m^{-1}.s^{-1}$

F Simulation : Depth-to-width impact on the rotating phenomena and control

We perform simulations to investigate the impact of the channel aspect ratio on the orbiting phenomena. The figure F.1 shows the rotating microrobot trajectories in the cross section of straight channels. The difference with a channel with an aspect ratio of one is that two orbiting center appear along the longest channel center lines instead of one in the center. For distance far too this point the trajectories orbit these two points. However in the vicinity of each of these points the microrobot orbit around a single point.

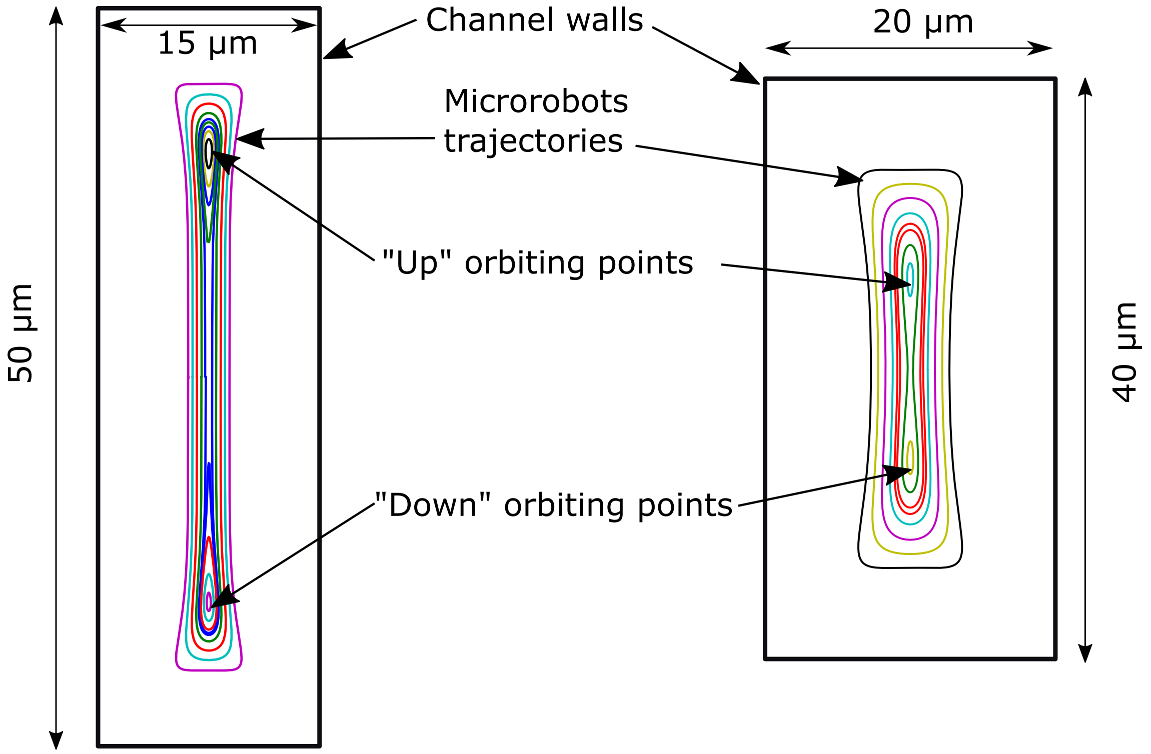

Figure F.1. Rotating phenomena in two channels with aspect ratio different from 1. In this case two orbiting center appear the longest center line.

However the control strategy in the paper is still possible in channels with aspect ratio different than 1. In this case the control put the robot in one of the two orbiting points. The figure F.2 shows an example where a relatively weak control of the pitch angle allow the control to switch between the two orbiting centers. One perspective of the existence of these two stable control point could be to select one direction during a channel bifurcation.

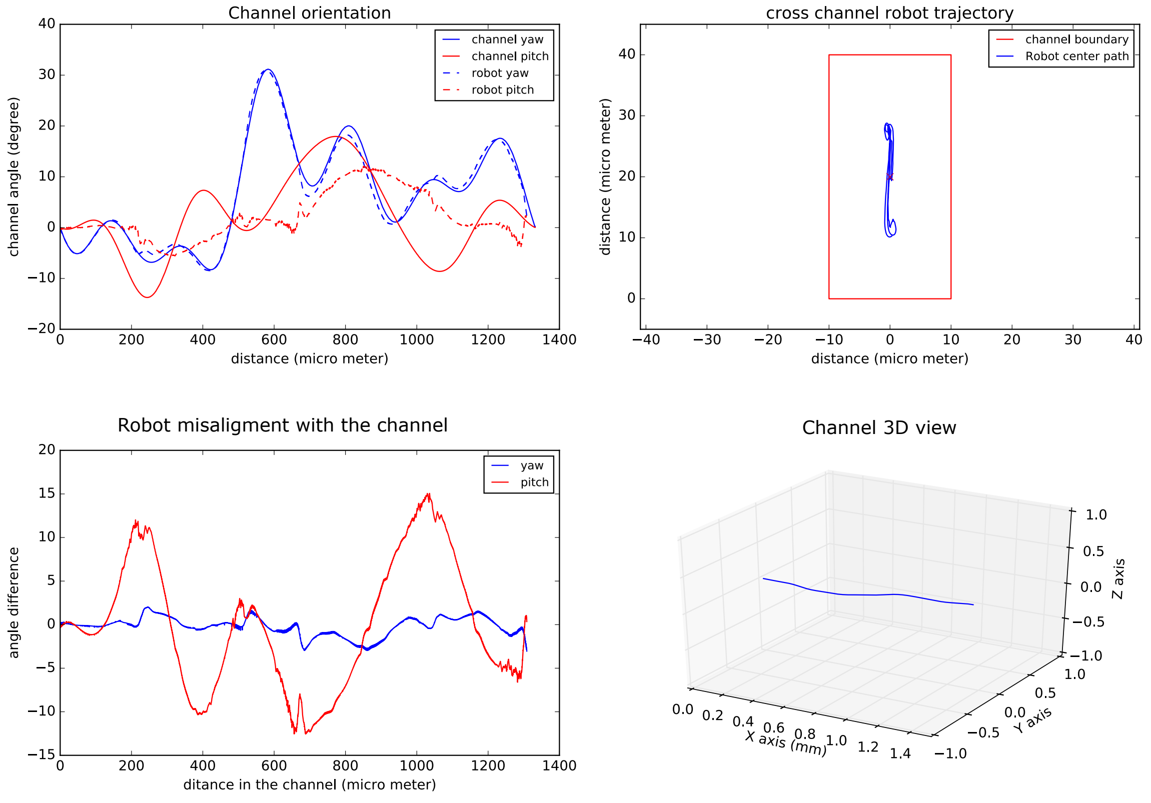

Figure F.2. Trajectory simulation in 20 μm to 40 μm rectangular section channel. Control parameters: ψ PID: $P = 0.04 Rad \cdot \mu m^{-1}$, $I = 0.01 Rad \cdot \mu m^{-1} \cdot s^{1}$,$D = 0 Rad \cdot \mu m^{-1} \cdot s^{-1}$; θ PID: $P = 0.02 Rad \cdot \mu m^{-1}$,$I = 0.1 Rad \cdot \mu m^{-1} \cdot s^{1}$,$D = 0.02 Rad \cdot \mu m^{-1} \cdot s^{-1}$

G Simulation: 3D Y-junction

The figure G.1 shows an example where the channel divide in two channels with a different pitch. In this example we used the 2D feedback) to control the robot in 3D with no knowledge on the channel pitch. We can see on the figure G.1 b) that after the junction the pitch angle of the microrobot converge to the pitch angle of the channel. As previously the 2D feedback is supposed to give a perpendicular image of the channel at any time. Before the junction the yaw angle is controlled to center the microrobot either to left or right of the channel and the pitch angle is controlled proportionally to the distance of the channel on x-axis. After the junction the 3D control based on the 2D feedback (feed with distance for the center on x-coordinate) is resume to center the robot in the channel without prior knowledge on the pitch angle of the channel.

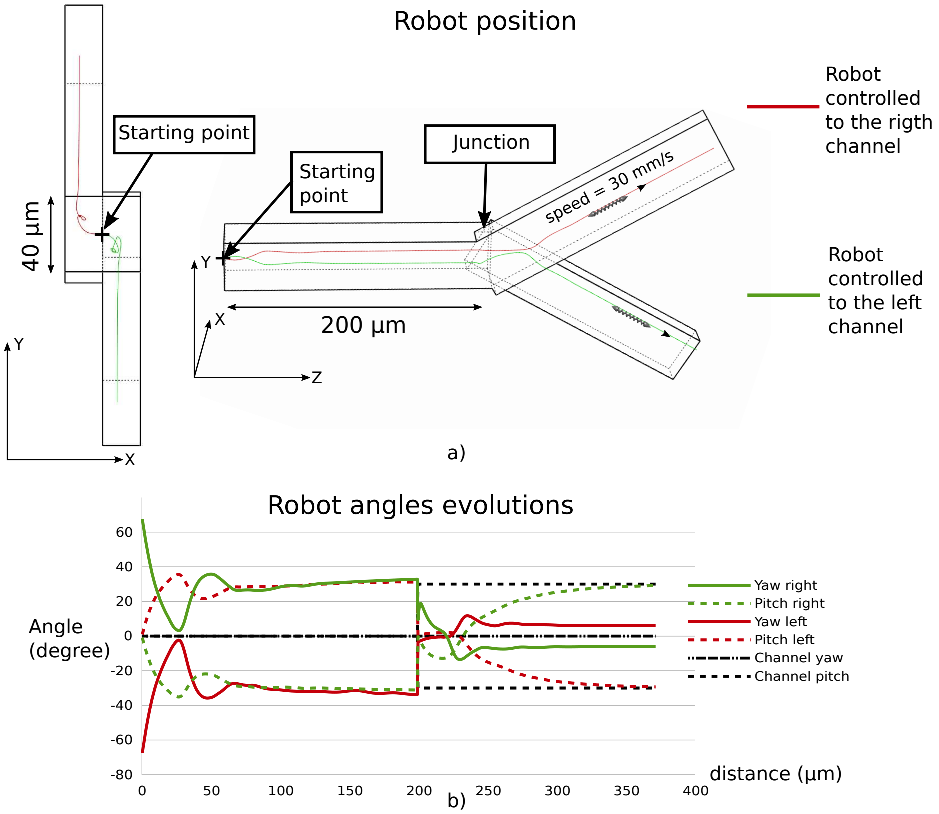

Figure G.1. Simulations of the helical microrobot trajectories through a Y type channel junction going out of planes. The main channel has a square section of 40 μm side. This channel splits in two similar rectangular section with 40 μm over 20 μm. a) Show trajectories base on a 2D image feedback control. b) Shows the evolution of the yaw and pitch angle on these two trajectories.

H Simulation manual

In the following we present a tutorial that will allow readers to reproduce the flow and trajectory simulations presented in this paper. This tutorial is proposed on a Linux Operating System (OS). This simulation could also work on Mac and Window but the command to launch FreeFem++ and python should be adapted. The simulations are split in two parts. The first part is the simulations of the flow created by the rotating microrobot in the 2D cross-section channel. These simulations are made with the software Freefem++ and are performed at different positions of the robot inside the channel. These positions are defined by a grid with constant spacing in the two directions between the node. When the flow simulations are finished, a file with csv format is generated. This file indicates the lateral drag and pressure force from the fluid on the robot for every positions on the grid. This code takes as input the dimension of the channel, the robot diameter (by assuming it is a cylinder), and the robot rotating frequency. The second part uses the generated file to simulate the robot trajectory on the channel. It consists of a Python script which applied the pseudo-code presented in the article. Therefore every time step it computes the new position of the robot on the 2D cross-section based on an interpolation of the flow simulation results. It also update the position along the channel to update its orientation changes. Note that for reading simplicity the pseudo-code in the article presents a Euler integration scheme whereas we use in the real code a 2nd order Runge Kutta scheme for better accuracy. In addition to the flow simulation results, the python code takes as input the speed of the robot generated by the helical propulsion at the frequency matching the one indicates in the flow simulations step. It also takes the lateral drag coefficient of the robot to convert the fluid lateral force to the lateral speed used to simulate the robot movements in the channel cross-section.

H.1 Tutorial: Trajectory simulation on a simple channel

Here is the protocol to perform simulations:

- Perform lateral drop test on the robot to get the lateral drag coefficient. The Stokeslet equation can also be used to get an experimental value without experimentation.
- Open flow_ cross_ section.edp with a text editor.
- Adjust the parameters for the simulation. If one changes the mesh resolution, the line 60 can be uncommented to display one mesh corresponding to one robot position. By default the radius of the robot is 2.5 μm, the channel as a section of 30μm, and the rotating speed is 100 Hz
- In a terminal type: FreeFem++ flow_ cross _ section.edp.
  You can install FreeFem++ on Linux by typing: sudo apt-get install FreeFem++
  For other systems please refer to the FreeFem++ web page.
  Note that this simulation takes times depending on the grid resolution. By default this grid is 50 by 50 and the simulation average time is 4 hours on an average desktop computer.
- When the simulation is finished open trajectory.py with a text editor.
- Edit the parameters to match the ones of the simulation. One also has to fill in the time step,, the lateral drag coefficient, the starting point positions, and the name of the output file generated by the flow simulation.
  If the default value does not change the flow simulation and trajectory should run and reproduce the trajectory of the helical microrobot presented in the paper on a 30 μm straight channel without any control and with a starting point distant from the center of 6μm. The time default time step is also set to match this particular case. This will reproduce trajectory similar to the one display in Figure 2 c)
- Run the simulation by typing: python trajectory.py.
  This simulation is quick (around 10 seconds)

H.2 Misalignement and control parameters

On control_ simulation.py file, the line 17 can be changed to set a constant misalignment of the robot with the channel. This can be used to reproduce simulation of Figure 3 d). Then a PID controller using Δy to control ψ (yaw angle) can be set on lines 22 to 24. By setting only a proportional gain ($PY = 0.25 \cdot 10^6$) the result of Figure 4 b) can be reproduced.

H.3 Generate 3D evolving channels

Finally, 3D evolving channels instead of straight channel can be simulated. This can be done by turning the value of the line 18 to "True". To adjust setting of this random generation use the line 315 and refer to line 208 for the function definition. Then another PID corrector using Δy to control θ (pitch angle) can be set on lines 27 to 29. By turning the two PID correctors ψ and θ, result from Figure 6 can be reproduced. For 30 μm channel dimension (default dimension) a working PID tuning is the following: ψ PID: $PY = 0.04 \cdot 10^6$,$IY = 0.01 \cdot 10^6$; θ PID: $PP = 0.02 \cdot 10^6$,$IP = 1 \cdot 10^6$,$IP = 0.02 \cdot 10^6$.

I Code source

I.1 flow_cross_section.edp

```
//----------------------------------LIBRARY----------------------------------------
//load "medit"
//----------------------------------PARAMETER--------------------------------------
//physical
real w = 100;                    // rotation speed of the robot (Rad.s^-1)
real nu = 2.37e-3;               // dynamic viscosity ( Pa.s) (isopropanol : nu =
    2.37e-3 ; see http://en.wikipedia.org/wiki/Viscosity#Dynamic_viscosity for other
     viscosity)
real speed = 100e-6;
real Rradius = 3e-6;     // robot radius
real Bradius = 30e-6;    //radius of the boundaries
real offsetx = 0;        //offset of the rts position with the center. 1 means
    touching the boundary
real offsety = 0;
//mesh resolution
real XYout = 200;        // resolution in XY 10 for quick compilation 50 for results
real XYin = 70;          // resolution in XY under the robot. 5 for quick
    compilation 30 for results
// Force calculation
int step =200; // number of simulation between the center of the channel and the
    side
int n =100; // number of points along the circle parameter to perform the
    calculation
real dr=0.2e-6; // distance from the perimeter to calculate the first order
    derivation of the speed.

//-----------------------------------OUTPUT FILE
    ------------------------------------------

ofstream out ("square_30.csv"); // for storring the force

//-----------------------------------GENERAL BOUNDARY DEFINITION
    ------------------------------

// number from 1 to 20 are associated with color
int robot = 10;
int doutsidefree = 15;
int goutsidefree = 20;

int kk= step;
int jj;

offsety=10e-6;

out << "step" <<";"<< step <<";"<<endl;
out << "x"<<";"<< "y"<<";"<< "Fx"<<";"<< "Fy"<<";"<< endl;
for (jj=round(-step/2);jj<round(step/2);jj++) //begin iteration on x
{
offsetx= Bradius *jj/round(step/2);
for (kk=round(-step/2);kk<round(step/2);kk++) // begin iteration on y
{
offsety= Bradius *kk/round(step/2);

//check if robot is inside the channel
```

```
if ((offsetx>-Bradius/2+Rradius+1e-6) && (offsetx<Bradius/2-Rradius-1e-6) && (
    offsety>Rradius+1e-6) && (offsety<Bradius-Rradius-1e-6) ) // 1 micrometer margin
{


//------------------------------------------------2D CONSTRUCTION
    ------------------------------------------------------------------



border S(t=-1,1){ x=Bradius*t/2; y=0;label=doutsidefree;}
border E(t=0,1){ x=Bradius/2; y=Bradius*t;label=doutsidefree;}
border N(t=1,-1){ x=Bradius*t/2; y=Bradius;label=doutsidefree;}
border W(t=1,0){ x=-Bradius/2; y=Bradius*t;label=doutsidefree;}

border b(t=0,2*pi){ x=Rradius*cos(t) + offsetx; y=Rradius*sin(t) + offsety;label=
    robot;}
//plot(S(20)+E(20)+N(20)+W(20)+b(30),wait=1) ;//to see a plot of the border mesh
//mesh Thwithouthole= buildmesh(a(XYout/2)+b(+XYin));
mesh Thwithhole = buildmesh(S(XYout/2)+E(XYout/2)+N(XYout/2)+W(XYout/2)+b(-XYin));
//plot(Thwithhole,wait=1,ps="Thwithouthole.eps");



//----------------------------------------------SAVE MESH
    --------------------------------------------------------------------------------
//savemesh(Thwithhole,"mesh2_cylindre_top.mesh");


//----------------------------------------------------PLOT--------------------------------------------------------------------------------


//----------------------------------------------------END MESH--------------------------------------------------------------------------
//----------------------------------------------------MAcro and function -----------------------------------------------------
 macro Grad(u) [dx(u),dy(u),dz(u)] // EOM
 macro div(u1,u2,u3) (dx(u1)+dy(u2)+dz(u3))  //EOM
 macro r(x,y) sqrt(x*x + y*y) //(sqrt(x*x + y*y))
 func real theta(real x, real y)
 {
        real outvar = 0.0;
        if (x>0)
        {
                outvar = atan(y/x);
        }
        if (x<0)
        {
                outvar = 3.1415 - atan(y/(-x));
        }
 return outvar;
 }

//----------------------------------------------------START SOLVING-------------------------------------------------------------------
fespace Uh(Thwithhole,P1b); Uh u,v,uu,vv;
fespace Ph(Thwithhole,P1); Ph p,pp;
solve stokes([u,v,p],[uu,vv,pp]) =
int2d(Thwithhole)(dx(u)* dx(uu)+dy(u)* dy(uu) + dx(v)* dx(vv)+ dy(v)* dy(vv)
```

```
+ dx(p)*uu + dy(p)*vv + pp*(dx(u)+dy(v))
- 1e-10*p*pp
)
+ on(robot,u=-w*(y-offsety) , v=w*(x-offsetx))
+ on(doutsidefree,u=0,v=0)
;


/*
//------------------------------------Streamline--------------------------------------------------------------
// Antoine's way
int k;

fespace Qh(Thwithhole,P1b);
Qh q,t;
solve streamlines(q,t) =
int2d(Thwithhole)(dx(q)*1*u*t+dy(q)*1*v*t
)
+ on(robot,q=0)
+ on(goutsidefree,q=y)
;

// standalone equation
/*
fespace Xh(Thwithhole,P1b);
Xh psi,phi;
solve streamlines(psi,phi) =
int2d(Thwithhole)( dx(psi)*dx(phi) + dy(psi)*dy(phi))
+ int2d(Thwithhole)( -phi*(dy(u)-dx(v)))
+ on(robot,psi=0)
+ on(outsidefree,psi=y)
;
*/
//medit("mm",Thwithhole,[u,v],order = 1,save="s

//-----------------------------------------------------WRITE SOLUTION
    -----------------------------------------------------------------------
/*
ofstream fu("u.txt");
fu << u[];
ofstream fv("v.txt");
fv << v[];
*/

//----------------------------------Plot--------------------------------------------------
// now work fine with medit : F1,F2,F3 to slice , m for intensity , w for vector

//medit("mm",Thwithhole,[u,v],order = 1,save="savemedit.solb");
string filename = "resultsquare/hemi_x="+offsetx+"_y="+offsety+".eps";
// speed
//plot([u,v],wait=0,value=true,coef=0.00005,ps=filename); // change wait to stop at
     each calculus (saving every plot version)
plot([u,v],wait=0,value=true,coef=0.00005,ps="buffer_square_30.eps"); // change
    wait to stop at each calculus (saving only last plot)
//plot(q,wait=1,value=true,coef=0.1,ps="it add it result.eps");
```

```
//inside variable
real[int] Fxtable;
real[int] Fytable;

real Fx = 0; // force on x
real Fy = 0; // force on y
real xdr,ydr,xx,yy; // x,y point xx,yy normal vector
real dun; // derivate along the normal
int dtheta;
//out << "dtheta*2*3.1415/n" <<";"<<"xx"<<";"<<"yy"<<";"<<"xdr"<<";"<<"ydr"<<";"<<
   "dun"  <<endl;
for(dtheta=0;dtheta<n;dtheta++)
        {
        xx = cos(dtheta*2*3.1415/n);
        yy = sin(dtheta*2*3.1415/n);
        xdr = (Rradius+dr) *xx +offsetx;
        ydr = (Rradius+dr) *yy +offsety;
        dun = (sqrt(u(xdr,ydr)*u(xdr,ydr) + v(xdr,ydr)*v(xdr,ydr)) - Rradius*w)/dr;
             // derivate = (speed(M(x,y) - speed(on the RTS) )/ (distance between
            the two)
        Fx = Fx + (-sin(dtheta*2*3.1415/n)) * Rradius*2*3.1415/n * nu * dun; //
            forcex = sum ( project_on_x(r d(theta) * viscosity* derivative)
        Fy = Fy + (+cos(dtheta*2*3.1415/n)) * Rradius*2*3.1415/n * nu * dun; //
            forcey = sum ( project_on_y(r d(theta) * viscosity* derivative)
        //out << dtheta*2*3.1415/n <<";"<<xx<<";"<<yy<<";"<<xdr<<";"<<ydr<<";"<<
            dun  <<endl;
        }
cout <<" Force on the RTS (at x ="<< offsetx<<"and y ="<<offsety<<")"<< "Fx ="<< Fx
    <<" Fy ="<< Fy <<endl;
out << offsetx<<";"<< offsety<< ";"<< Fx<<";"<< Fy<<";"<< endl;
}// end if inside the the channel
else
{
out << offsetx<<";"<< offsety<< ";"<< 0<<";"<<0<<";"<< endl;
}
}// end iteration on offsetx
}// end iteration on offsety
cout << "END OF ITERATION" << endl;
```

I.2 control_ simulation.py

```
#--- Python library ---
import csv
import numpy as np
import matplotlib.pyplot as plt
from mpl_toolkits.mplot3d import Axes3D
import math as mt
from numpy.fft import *
plt.ion() #desactivate interactive mode for plot
#--- parameters ---
#simulation
file_name='square_30.csv' #input file with flow simulation result (output of
    Freefem++)
e=30e-6 # channel side wall lenght
dist_simu=2000e-6 # in meter, length of the channel for the simulation
step=0.05 # in second, time step for the simulation
a=8.4e-7/42e-6 #s-1*N-1 (horizontal a divide by the length of the robot);
    correspond o the drag coeficient of the RTS
P0=np.array([0,20e-6]) # starting point of the simulation
speed=30e-6 #robot longitudinal speed in m/s
#parameters
K1=100000 # rad/m yaw proportional corrector
K2=100000 # rad/m pitch proportional corrector
K3=100000 # rad/m/s pitch integral corrector

#--- calculated constant ---
time_simu=dist_simu/speed
nb_iter=int(time_simu/step);


#--- import result file from Freefem simulation ---
with open(file_name, 'rb') as csvfile:
    spamreader = csv.reader(csvfile, delimiter=';', quotechar='|')
    spam=spamreader.next() #get step
    t_size=int(spam[1])
    spamreader.next() #skip second line
    k=1
    X=[]
    Y=[]
    FX=[]
    FY=[]
    XX=[]
    YY=[]
    FFX=[]
    FFY=[]
    for row in spamreader:
        #print(row)
        X.append(float(row[0]))
        Y.append(float(row[1]))
        FX.append(float(row[2]))
        FY.append(float(row[3]))
        if  k == t_size:
            XX.append(X)
            YY.append(Y)
            FFX.append(FX)
```

```
                FFY.append(FY)
                X=[]
                Y=[]
                FX=[]
                FY=[]
                k=0
            k=k+1
    X=(np.array(XX))
    Y=(np.array(YY))
    FX=(np.array(FFX))
    FY=(np.array(FFY))



#--- Functions definition for the simulation ---
def force_at_xy (x,y,gx,gy,X,Y,FX,FY):
    """ interpolate the force on the robot at the given location (x,y)"""
    for xx in range(1,X.shape[0],1):
        for yy in range(1,X.shape[1],1):
            if ((X[xx][yy]>x) and Y[xx][yy]>y): #detect the firs time above, it
               mean xx,yy are the coordinate at the top right corner of the grid
               containing the point (x,y)
                dx=(x-X[xx-1][yy])/(X[xx][yy]-X[xx-1][yy])
                dy=(y-Y[xx][yy-1])/(Y[xx][yy]-Y[xx][yy-1])
                #print(dx)
                #calculate intermdiate point on xx-1 and xx line
                fx1=FX[xx-1][yy-1]*(1-dy)+FX[xx-1][yy]*(dy)
                fy1=FY[xx-1][yy-1]*(1-dy)+FY[xx-1][yy]*(dy)
                fx2=FX[xx][yy-1]*(1-dy)+FX[xx][yy]*(dy)
                fy2=FY[xx][yy-1]*(1-dy)+FY[xx][yy]*(dy)
                #calculae final point
                fx=fx1*(1-dx)+fx2*dx
                fy=fy1*(1-dx)+fy2*dx
                F=np.array([fx+gx,fy+gy])
                return(F)
    F=np.array([0,0])
    return(F)

def speed_at_xy (x,y,gx,gy,X,Y,FX,FY,a):
    """a the drag coeficient such as Fdrag=speed*a. An other experiment need to be
       made for this a because it is the drag coeficient for the RTS moving
       othogonal o the flow"""
    F=force_at_xy(x,y,gx,gy,X,Y,FX,FY)/a
    return F


def RK2 (step,x,y,gx,gy,X,Y,FX,FY,a):
    """range kutta 2: return the position of the robot after the time step
    here we assume that the force is directly proportional (egal for now) to the
       displacement as the inertia of the robot is negligible and the viscosity
       directly compensate this move
    step: step in second
    x,y position of the robot compare to the channel center
    gx,gy lateral force on the robot
    X,Y,FX,FY Force data from the freefem++ simu
```

```
    a: lateral friction coef
    """
    p0=np.array([x,y])
    #print("1")
    dp=speed_at_xy(x,y,gx,gy,X,Y,FX,FY,a)
    #print(dp)
    """ half disance calculation """
    p12=p0+dp*step/2
    #print("2")
    dp12=speed_at_xy(p12[0],p12[1],gx,gy,X,Y,FX,FY,a)
    #print(dp12)
    """point calculation"""
    p1=p0+dp12*step
    return p1



def smoother (signal,degree):
   """smooth a signal with fft, degree =0 is no smoothing, degree = 1 is nul signal
       (used to smooth the random generated channel)"""
   fsignal = rfft(signal)
   hsize= int(len(signal)/2.0)
   cut=int(hsize*degree)
   for n in range(hsize-cut,len(fsignal)-1):
       fsignal[n]=0
   ssignal=irfft(fsignal)
   return ssignal


class PID:
    """A PID corrector Class : tested"""
    def __init__(self):
        self.int_error=0;
        self.last_error=0;
        self.P=0
        self.I=0
        self.D=0
        self.last_time=0
    def tuning(self,set_P,set_I,set_D):
        self.P=set_P
        self.I=set_I
        self.D=set_D
    def next_point(self,error,time):
        #proportional gain
        pv=self.P *error
        # Integral gain
        self.int_error = self.int_error + (time-self.last_time)*error
        iv = self.I * self.int_error
        #Derivative Gain
        diff=(error-self.last_error)/(time-self.last_time)
        dv= self.D * diff
        # update
        self.last_error=error
        self.last_time=time
        return (pv+iv+dv)
```

```python
    def reset(self):
        self.int_error=0;
        self.last_error=0;
        self.last_time=0

class Robot:
    def __init__(self):
        self.x=0
        self.y=0
        self.yaw=0
        self.pitch=0

class Channel:
    def __init__(self):
        self.z=np.array
        self.yaw=np.array
        self.pitch=np.array
    def create_channel_rdm(self,dist,resolution,kyaw,kpitch,smooth):
        """
        Create_channel: create a random generated channel. It return a  dist/
            resolution*2 matrix of the evolution of the pitch and yaw angle.
        dist and resolution are in micron. Kyaw and kpitch are the change rate of
            the channel directions. In one micrometer the channel's direction change
             of
        a value homogenously distibute between Kyaw (or Kpitch) and 0.
        """
        lz=[0]
        lyaw=[0]
        lpitch=[0]
        for k in range(1,int(dist/resolution),1):
            lz.append(k*resolution)
            lyaw.append(lyaw[-1]+kyaw*(np.random.random_sample()-0.5) )
            lpitch.append(lpitch[-1]+kpitch*(np.random.random_sample()-0.5) )
        #smooth
        self.yaw=np.array(smoother(lyaw,smooth))
        self.pitch=np.array(smoother(lpitch,smooth))
        self.z=np.array(lz)
        #set 0 at origin
        self.yaw=self.yaw-self.yaw[0]
        self.pitch=self.pitch-self.pitch[0]

    def create_channel_linevol(self,dist,resolution,yaw,pitch):
        """
        Create_channel: create a random generated channel. It return a  dist/
            resolution*2 matrix of the evolution of the pitch and yaw angle.
        dist and resolution are in micron. Kyaw and kpitch are the change rate of
            the channel directions. In one micrometer the channel's direction change
             of
        a value homogenously distibute between Kyaw (or Kpitch) and 0.
        """
        lz=[0]
        lyaw=[0]
        lpitch=[0]
        for k in range(1,int(dist/resolution),1):
            lz.append(k*resolution)
```

```
            lyaw.append((1-(k/(dist/resolution)))*yaw[0] + (k/(dist/resolution))*
                yaw[1])
            lpitch.append((1-(k/(dist/resolution)))*pitch[0] + (k/(dist/resolution)
                )*pitch[1])
        #smooth
        self.yaw=np.array(lyaw)
        self.pitch=np.array(lpitch)
        self.z=np.array(lz)
        #set 0 at origin
        self.yaw=self.yaw-self.yaw[0]
        self.pitch=self.pitch-self.pitch[0]

    def plot_channel(self):
        plt.figure(1)
        plt.subplot(221)
        plt.plot(self.z*1e6,self.yaw*180/3.1415,'r')
        plt.plot(self.z*1e6,self.pitch*180/3.1415,'b')
        plt.legend(('yaw', 'pitch'))
        plt.ylabel('channel angle (degree)')
        plt.xlabel('distance (micro meter)')
        plt.title('Channel orientation')
        plt.show()
        #ax = fig.add_subplot(111, projection='3d')
        #ax.plot(channel[0,:],channel[1,:],channel[2,:])
        #plt.show()
    def get_yaw_at_position(self,dist):
        """give the value of yaw at a given distance"""
        buff=self.z.searchsorted(dist)
        return(self.yaw[buff])
    def get_pitch_at_position(self,dist):
        """give the value of pitch at a given distance"""
        buff=self.z.searchsorted(dist)
        return(self.pitch[buff])
    def plot_channel_3D(self):
        """To plot a channel in 3D"""
        #Calculate x,y,z coordinate
        x=[0]
        y=[0]
        z=[0]
        dist=self.z[1]-self.z[0] # ONLY WORK FOR REGULAR DISTANCE
        for k in self.z:
            yaw=self.get_yaw_at_position(k)
            pitch=self.get_pitch_at_position(k)
            vect=np.array([mt.cos(pitch)*mt.sin(yaw),mt.sin(pitch),mt.cos(pitch)*mt
                .cos(yaw)])
            x.append(x[-1] + vect[0]*dist)
            y.append(y[-1] + vect[1]*dist)
            z.append(z[-1] + vect[2]*dist)


        fig = plt.figure(1)
        ax = fig.add_subplot(224, projection='3d')
        ax.plot(x, y, z)
        ax.set_xlabel('X axis')
        ax.set_ylabel('Y axis')
```

```python
        ax.set_zlabel('Z axis')
        plt.show()

#--- START THE SIMULATION ---
#Object definition
channel = Channel()
channel.create_channel_rdm(dist_simu,1e-6,0.08,0.08,0.99) # Create a random channel
#channel.create_channel_linevol(dist_simu,1e-6,[0,0],[0,360*3.1415/180]) #create a
   looping channel
channel.plot_channel()
yaw_corrector= PID()
pitch_corrector=PID()
yaw_corrector.tuning(K1,0,0)
pitch_corrector.tuning(K2,K3,0)

robot = Robot()
robot.x=P0[0]
robot.y=P0[1]

#----main loop----
plt_rx=[]
plt_ry=[]
plt_ryaw=[]
plt_rpitch=[]
plt_z=[]
plt_rx.append(robot.x)
plt_ry.append(robot.y)
plt_z.append(0)
for k in range(1,nb_iter,1):
    #diff between robot and channel
    time =k*step
    dyaw=robot.yaw-channel.get_yaw_at_position(time*speed)
    dpitch=robot.pitch-channel.get_pitch_at_position(time*speed)
    #get force on x axis = yaw and y axis = pitch
    fx = speed*mt.sin(dyaw)*a
    fy = speed*mt.sin(dpitch)*a
    # simulate robot next point
    P=RK2(step,robot.x,robot.y,fx,fy,X,Y,FX,FY,a)
    robot.x=(P[0])
    robot.y=(P[1])
    #Control:
    # assume we know only: robot.x robot.yaw channel.yaw
    robot.yaw=yaw_corrector.next_point(-robot.x,time) + channel.get_yaw_at_position
       (time*speed) # robot.yaw is set at the same that the channel + a correction
       proportional to the x distance between the robot and the center
    robot.pitch=pitch_corrector.next_point(robot.x,time)
    #storring for plot
    plt_rx.append(robot.x)
    plt_ry.append(robot.y)
    plt_ryaw.append(robot.yaw)
    plt_rpitch.append(robot.pitch)
    plt_z.append(time*speed)


#----plot results----
```

```
#convert list to numpy
plt_rx = np.array(plt_rx)
plt_ry = np.array(plt_ry)
plt_ryaw = np.array(plt_ryaw)
plt_rypitch = np.array(plt_rpitch)

plt.figure(1)
plt.subplot(222)

plt.ylabel('distance (micro meter)')
plt.xlabel('distance (micro meter)')
plt.title('cross channel robot trajectory')
plt.show()
xb=np.array([-e/2,e/2,e/2,-e/2,-e/2])
yb=np.array([0,0,e,e,0])
xb=xb
yb=yb
plt.plot(xb*1e6,yb*1e6) # channel
plt.plot(plt_rx*1e6,plt_ry*1e6) # trajectory
plt.plot(plt_rx[0]*1e6,plt_ry[0]*1e6,'xr') # starting point
plt.axis('equal')
plt.axis([(-e/2-5e-6)*1e6,(e/2+5e-6)*1e6,(-5e-6)*1e6,(e+5e-6)*1e6])
plt.legend(('channel boundary','Robot center path'))

plt.show()

def square(list):
    return map(lambda x: mt.sqrt(x), list)

plt.subplot(223)
plt.plot(np.array(plt_z)*1e6,np.array(square(plt_rx*plt_rx+(plt_ry-e/2)*(plt_ry-e
    /2)))*1e6)
plt.xlabel('disantance in the channel (micro meter)')
plt.ylabel('distance too the channel center (micro meter)')
channel.plot_channel_3D()
plt.ioff() #resactivate interactive mode for plot
plt.show()
```
